# Case Report: Beyond the monophasic myth: relapsing Baló’s concentric sclerosis with residual disability

**DOI:** 10.3389/fnhum.2026.1794003

**Published:** 2026-03-25

**Authors:** Weiya Li, Zhao Jin, Chensijia Zhang, Lele Wang, Mengyi Zhao, Jing Zhang, Qian Li

**Affiliations:** 1 Department of Neurology, Affiliated Nankai Hospital of Tianjin Medical University (Tianjin Nankai Hospital), Tianjin, China; 2 Hospital of Integrated Chinese and Western Medicine, Tianjin University, Tianjin, China; 3 Hospital of Integrated Chinese and Western Medicine, Tianjin University of TCM, Tianjin, China; 4 Tianjin Key Laboratory of Acute Abdomen Disease Associated Organ Injury and ITCWM Repair, Tianjin, China; 5 Institute of Integrative Medicine for Acute Abdominal Diseases, Tianjin, China; 6 Department of Neurology, Tianjin University Central Hospital (The Third Central Hospital of Tianjin), Tianjin, China; 7 The School of Medicine, Nankai University, Tianjin, China; 8 Tianjin Medical University, Tianjin, China

**Keywords:** Baló’s concentric sclerosis, corticosteroids, hypertension, MRI, relapse

## Abstract

**Background:**

Baló’s concentric sclerosis (BCS) is a rare demyelinating disorder with a striking radiological signature. Its potential for relapse and the impact of vascular comorbidities on disease course remains underrecognized.

**Case presentation:**

A 52-year-old woman presented with acute limb weakness and sensory disturbances. MRI revealed pathognomonic concentric rings with concentric ring-like enhancement. Despite initial remarkable response to high-dose corticosteroids, she experienced a relapse at 10 months with new-onset limb tremor, persistent blood pressure fluctuations (on the basis of partial recovery) and lesion evolution on MRI. Repeat steroid therapy achieved disease stabilization, but residual motor disability persisted with a 1-year total follow-up (6 months of stable condition after relapse).

**Conclusion:**

This case highlights BCS’s relapsing potential and the synergistic effect of uncontrolled hypertension on disease progression, challenging the notion of its uniformly monophasic course. Vigilant monitoring of vascular risk factors and personalized rehabilitation strategies are crucial, even after excellent initial recovery.

## 1 Introduction

Baló’s concentric sclerosis (BCS) stands as one of the most radiographically dramatic entities in neuroinflammatory disorders (Etemadifar et al., 2025; Peraza et al., 2025). While classically defined by its signature “onion-bulb” pattern on MRI, the clinical course of BCS remains enigmatic (Thomas et al., 2024; Kchaou et al., 2019) and awareness of this rare disorder remains limited across clinical and allied health professions (Ananya et al., 2022). Once considered predominantly monophasic, emerging evidence from recent case series and literature reviews (Etemadifar et al., 2025; Peraza et al., 2025; Fonseca et al., 2024) suggests a more heterogeneous spectrum, including relapsing forms with accumulating disability (Luan et al., 2025; Zhang et al., 2024) and even atypical presentations that overlap with other neurological conditions (Nouri et al., 2022). This evolving understanding challenges simplistic classifications and demands closer examination of its long-term trajectory.

We present a case of a middle-aged woman with typical BCS imaging who, despite robust initial treatment response, experienced a delayed relapse featuring new-onset motor symptoms and persistent blood pressure fluctuations. This case highlights critical gaps in our current management paradigms, particularly the lack of attention to vascular comorbidity management in BCS patients, and underscores the need for vigilant, long-term monitoring in this rare disorder. This study reports a case of relapsing BCS with uncontrolled hypertension and residual motor disability, analyzes its clinical and imaging characteristics, discusses the impact of vascular comorbidities on disease relapse and the treatment strategy for relapsing BCS, and aims to provide clinical insights for the long-term monitoring and personalized management of relapsing BCS.

## 2 Case presentation

### 2.1 Initial presentation and diagnostic journey

A 52-year-old woman (Ms. W) was admitted in March 2024 with a 13-day history of left lower limb numbness and weakness. Within one week, her symptoms progressed to generalized limb weakness rendering her unable to walk, accompanied by a distinctive chest band-like sensory disturbance at the T4–T6 spinal segment, manifested as numbness and tightness that aggravated with trunk movement, a typical sensory symptom in inflammatory demyelinating diseases including BCS. Her medical history included untreated hypertension for 3 years (baseline blood pressure 160–170/90–100 mmHg), and long-term uncontrolled hypertension may reduce cerebral perfusion and motor reserve, and also increase the risk of inflammatory activity and lesion progression in BCS by inducing vascular endothelial dysfunction and remote gastrointestinal polyp surgery.

Neurological examination revealed asymmetric weakness, [left upper limb grade 3/5, left lower limb grade 2/5, Medical Research Council (MRC) scale], decreased tone, bilateral positive Babinski signs, and impaired coordination. Routine laboratory investigations and CSF analysis were unremarkable, with negative demyelinating antibodies and absent oligoclonal bands.

### 2.2 Clinical timeline

To clarify the temporal sequence of symptom onset, diagnosis, treatment, and disease progression in this relapsing BCS case, the entire clinical course from initial symptom appearance to follow-up after relapse is summarized in Table 1.

**Table 1:** Clinical timeline of the patient with relapsing Baló’s concentric sclerosis.

Time	Duration of disease (days)	Clinical event	Clinical/Examination description
25/February/2024	Day 0	Onset of initial symptoms	Left lower limb numbness and weakness without obvious predisposing factors
01/March/2024	Day 6	First brain MRI examination	Brain MRI at an external hospital showed demyelination-like signal abnormalities in the right centrum semiovale and left parietal lobe, suggesting a possible demyelinating lesion
04/March/2024	Day 8	Enhanced brain MRI examination	Enhanced brain MRI at an external hospital revealed characteristic “onion-bulb” concentric ring-like enhancing lesions, which highly suggested Baló’s concentric sclerosis (BCS) in combination with clinical manifestations
08/March/2024	Day 13	Hospital admission	Admitted due to progressive aggravation of limb weakness and inability to walk independently; accompanied by chest band-like sensory disturbance and bilateral positive Babinski signs
10/March/2024	Day 14	Initiation of treatment	Started high-dose methylprednisolone (500 mg/day) pulse therapy for 3 consecutive days
20/March/2024	Day 19	Hospital discharge	Discharged after partial improvement of motor function (left upper limb muscle strength MRC grade IV, left lower limb muscle strength MRC grade III)
Early May 2024	Day 66	Rehabilitation milestone	Regained independent walking ability after 1.5 months of standardized rehabilitation treatment, achieving complete remission of motor function
13/January/2025	10.5 months after initial onset	Disease relapse	Developed new-onset left upper limb tremor, persistent numbness of bilateral lower limbs, and spontaneous blood pressure fluctuations
15/January/2025	2 days after relapse	Relapse treatment and reexamination	Initiated the second course of corticosteroid therapy; rechecked brain MRI showed that the original subcortical white matter lesions progressed to more homogeneous plaque-like changes
25/February/2025	12 months after initial onset	Follow-up	Clinical symptoms were stable, with residual left limb weakness; occasional use of a walking aid was required for ambulation

### 2.3 Diagnostic imaging and acute management

**Figure 1:**
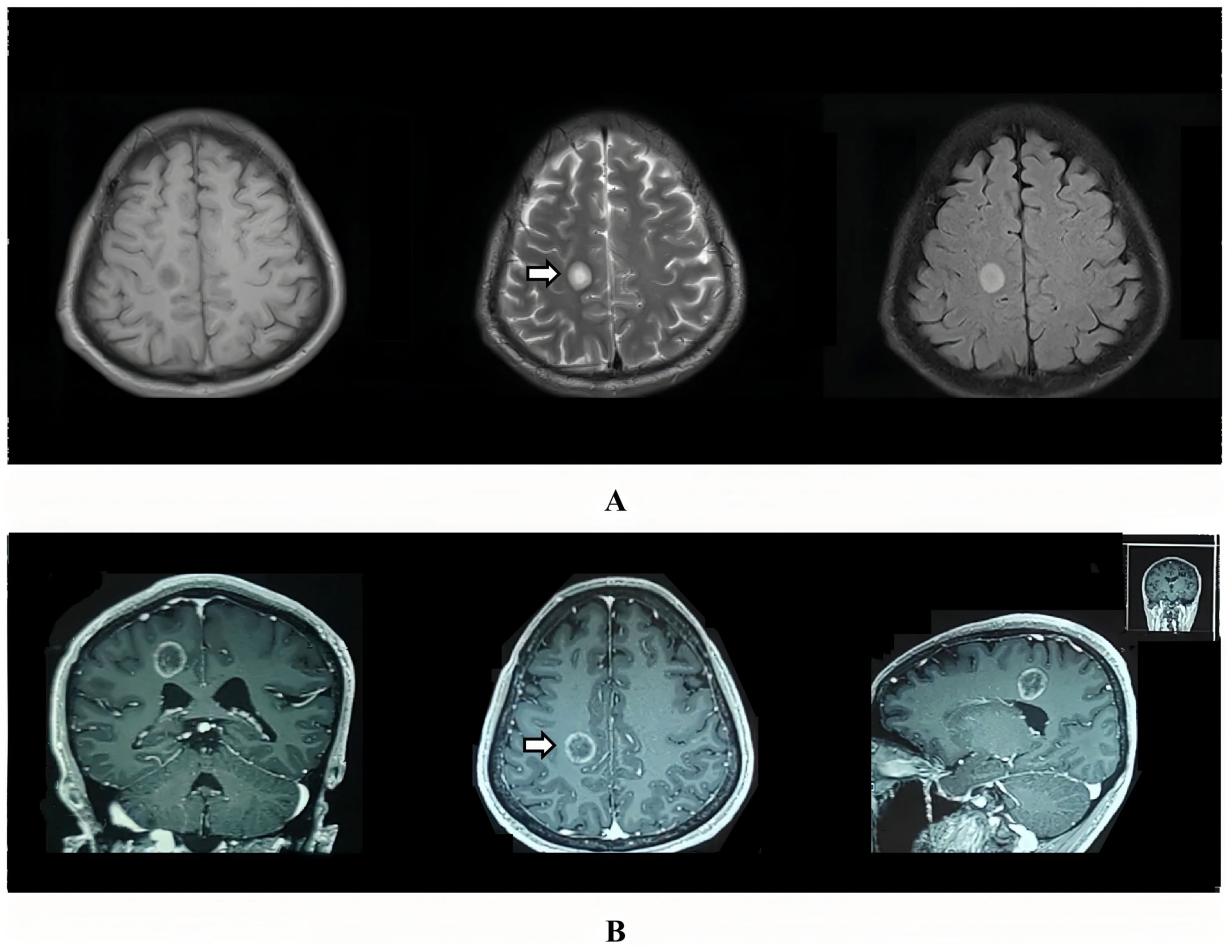
Serial brain MRI findings. **(A)** Axial T1-weighted, T2-weighted, and FLAIR sequences demonstrated demyelination-like signal abnormalities in the right centrum semiovale and left parietal lobe. White arrows indicate the core demyelinating lesions. **(B)** Gadolinium-enhanced coronal, axial, and sagittal T1-weighted images revealed characteristic “onion-bulb” concentric ring-like enhancing lesions, featuring alternating T2/FLAIR hyperintense-isointense rings (consistent with the “fried egg” sign in Balo’s concentric sclerosis). White arrows indicate the pathognomonic concentric ring lesions.

Brain MRI performed on day 6 showed suggestive demyelinating lesions. A subsequent enhanced MRI on day 8 demonstrated the definitive “onion-bulb” appearance—concentric T2/FLAIR rings with characteristic concentric ring-like enhancement (Figure 1), confirming BCS diagnosis.

The patient received high-dose methylprednisolone (500 mg/day for 3 days), resulting in meaningful motor improvement. She was discharged on a tapering steroid regimen with adjunctive medications, and regained independent walking ability after 1.5 months of dedicated rehabilitation.

### 2.4 Unexpected relapse and management

**Figure 2:**
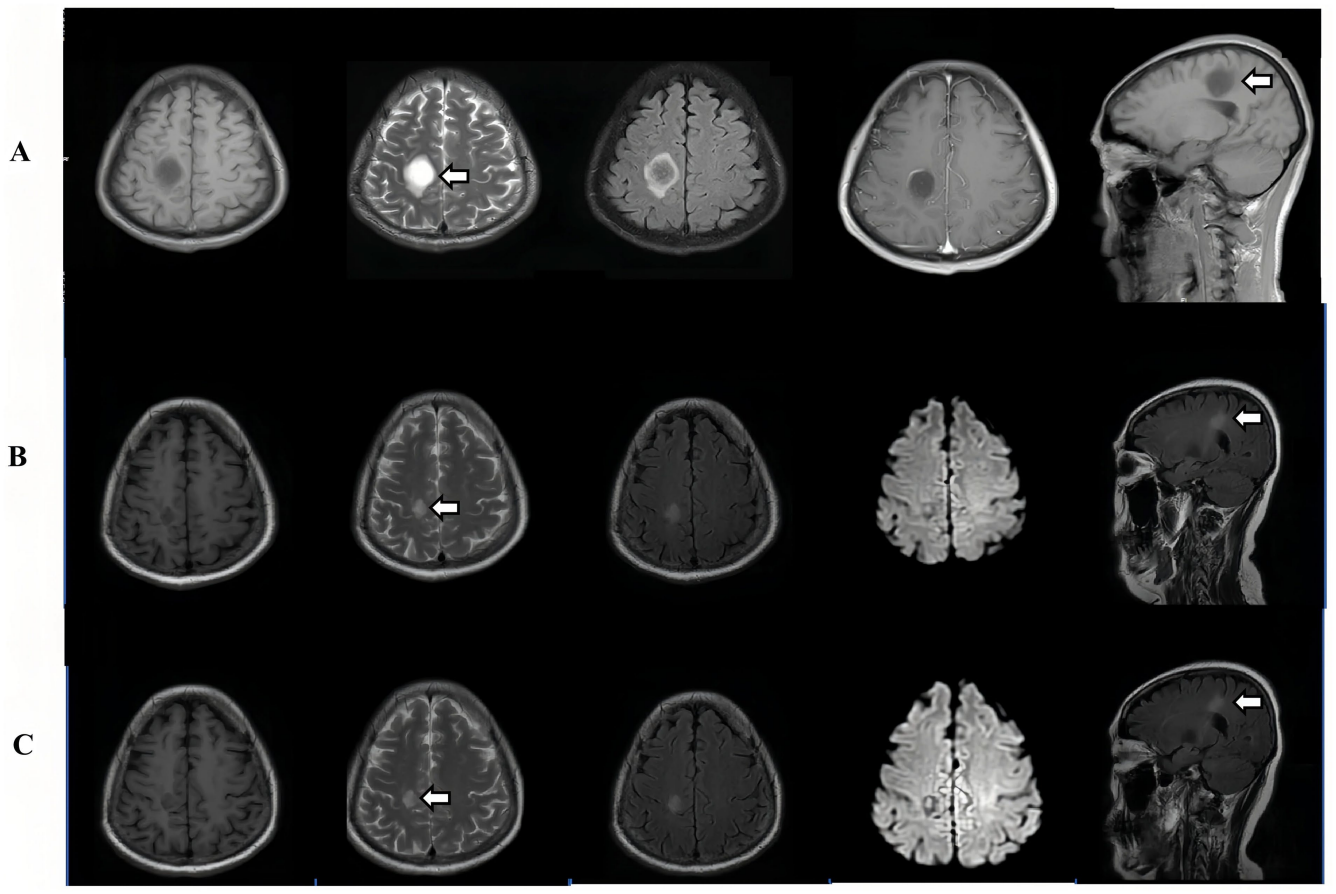
Temporal evolution of brain lesions on MRI. **(A)** (March 2024) Multisequence brain MRI (T1-weighted, T2-weighted, FLAIR, and gadolinium-enhanced sequences) showed a prominent lesion in the right cerebral hemisphere, with characteristic signal abnormalities and ring-like contrast enhancement (white arrow indicates the core lesion at initial onset). **(B)** (October 2024) Follow-up MRI demonstrated partial resolution of the lesion, with reduced signal heterogeneity and diminished contrast enhancement (white arrow indicates the partially resolved lesion after treatment). **(C)** (January 2025) The latest imaging revealed further evolution, with the lesion transforming into a more homogeneous plaque and marked reduction of contrast enhancement, indicating disease progression and modulation over time (white arrow indicates the evolved lesion at relapse).

Ten months later, the patient represented with persistent bilateral lower limb numbness, left upper limb tremor, and newly documented blood pressure fluctuations. Follow-up MRI showed evolution of previous lesions into more homogeneous plaques with reduced enhancement (Figure 2).

A second corticosteroid course effectively stabilized her symptoms. Intravenous corticosteroid was chosen as the first-line therapy for the relapse because: (1) the patient achieved a significant response to initial steroid therapy, indicating a good steroid sensitivity; (2) plasmapheresis, IVIG and disease-modifying therapies (DMTs) lack high-quality clinical evidence for relapsing BCS, and there is no unified clinical guideline for the use of these therapies in BCS; (3) the patient had no severe contraindications to corticosteroids and refused aggressive immunotherapy due to concerns about adverse reactions. At one-year follow-up (6 months of stable condition after relapse), she maintained clinical stability for over six months, though residual left-sided weakness necessitated the use of a walking aid.

## 3 Discussion

Baló’s concentric sclerosis (BCS) represents a rare and radiologically striking variant within the spectrum of inflammatory demyelinating diseases of the central nervous system. The case of a 52-year-old female presenting with acute limb weakness, sensory issues, and a chest band-like sensation offers a window into the clinical characteristics of BCS. Analyzing her clinical journey with 1-year total follow-up (6 months of stable condition after relapse) helps crystallize several key insights, which we distill into three core teaching points.

Key teaching points:

- BCS can follow a relapsing course despite characteristic imaging and initial treatment response, especially in patients with incomplete initial recovery.
- Uncontrolled vascular comorbidities (e.g., hypertension) may exacerbate disease progression and increase the risk of BCS relapse.
- Long-term motor disability can persist even after clinical stabilization of relapsing BCS, requiring personalized rehabilitation.

The 10-month relapse in our patient, featuring new-onset limb tremor and persistent blood pressure fluctuations, challenges the traditional view of BCS as a uniformly monophasic illness (Martinez et al., 2021; Tzanetakos et al., 2020). This temporal pattern suggests the disease may have a more protracted inflammatory activity than previously recognized. The subacute onset of new motor symptoms months after the initial event implies that this case may suggest that a state of clinically silent yet biologically active pathology could exist in BCS, which is an inference based on this single case. Hypothetically, persistent oligodendrocyte injury and microglial activation might drive the ongoing inflammatory activity, though secondary neurodegeneration and vascular comorbidities could also contribute to the disease progression. Notably, the above pathophysiological inferences are based on this single BCS case and existing literature, and other potential mechanisms cannot be excluded, such as secondary neurodegeneration induced by initial demyelination and impaired cerebral perfusion associated with long-term hypertension, which may also participate in the disease relapse and motor symptom exacerbation. The immunological profile of our patient (negative AQP4-IgG, MOG-IgG, and OCBs) aligns with BCS as a distinct entity from classic MS or NMOSD (Mei et al., 2025; Kira, 2025; Elias et al., 2025; Sharifi et al., 2024). With 1-year total follow-up (6 months of stable condition after relapse), the precise mechanisms driving disease recurrence remain elusive, though this serological signature may reflect the unique pathogenesis of pattern III oligodendrocytopathy (Sawczynska et al., 2025; Anschel, 2006; Wiendl et al., 2005).

### 3.1 Differential diagnosis of BCS in the inflammatory demyelinating disease spectrum

As a rare variant of central nervous system inflammatory demyelinating disease, BCS requires differentiation from other disorders presenting with tumefactive demyelinating lesions, primarily tumefactive multiple sclerosis (TMS) and MOG antibody-associated disease (MOGAD). Key discriminative features include radiological, clinical, and serological characteristics: BCS is distinguished by concentric ring-like enhancement (onion-bulb sign) on imaging, rare occurrence of Dawson’s fingers (periventricular demyelinating lesions), and a predominantly monophasic course with lower relapse rates (Sawczynska et al., 2025; Jolliffe et al., 2021), whereas TMS typically exhibits open-ring enhancement, frequent Dawson’s fingers, higher relapse risk, and greater tendency to progress to classic multiple sclerosis. In contrast, MOGAD is characterized by predominant optic neuritis and myelitis (with extremely rare concentric ring-like lesions), steroid-dependent relapses, infrequent progressive motor dysfunction with tremor (Mei et al., 2025; Kamari et al., 2023b), and positive MOG-IgG serology—distinct from BCS, where most patients are negative for demyelinating antibodies (including AQP4-IgG and MOG-IgG), consistent with our patient’s immunological profile. These differential points facilitate early and accurate diagnosis of BCS and inform the development of individualized treatment strategies.

### 3.2 Impact of uncontrolled hypertension on relapsing BCS

Uncontrolled hypertension is an important modifiable risk factor that exacerbates the disease course of relapsing BCS, and its potential mechanisms are mainly reflected in three aspects: (1) Vascular endothelial dysfunction: Long-term elevated blood pressure induces damage to cerebral vascular endothelial cells, increases blood–brain barrier permeability, and promotes the infiltration of inflammatory factors into the central nervous system, thereby sustaining and aggravating the inflammatory demyelinating process of BCS. (2) Impaired oligodendrocyte regeneration: Hypertension reduces cerebral perfusion and the supply of oxygen and nutrients to oligodendrocytes, inhibits the proliferation and differentiation of oligodendrocyte precursor cells, and impairs myelin repair, leading to incomplete initial recovery and increased relapse risk. (3) Synergistic damage to motor pathways: Hypertensive cerebral small vessel disease causes microinfarction and white matter lesions in the motor cortex and corticospinal tract, which synergizes with BCS-related demyelinating lesions to exacerbate motor symptoms (e.g., limb weakness, tremor) and increase the risk of residual motor disability. In this case, the patient’s 3-year history of untreated hypertension was closely associated with the occurrence of relapse and the persistence of motor symptoms, which suggests that strict vascular risk factor control should be incorporated into the routine management of BCS patients.

### 3.3 Association between blood pressure fluctuations and autonomic dysfunction in BCS

The blood pressure fluctuations and periumbilical paroxysmal involuntary twitching observed in this patient during relapse are manifestations of autonomic dysfunction, which may be related to BCS involving the autonomic nervous pathway. Existing studies have shown that MS/BCS spectrum diseases can damage the autonomic nervous center in the brainstem and spinal cord, leading to abnormalities in blood pressure regulation, gastrointestinal motility and other functions (Kira, 2025; Elias et al., 2025). The autonomic dysfunction in this case is not only related to long-term uncontrolled hypertension-induced vascular endothelial damage, but also may be caused by the involvement of autonomic nervous fibers in the demyelinating lesions of BCS. This indicates that autonomic dysfunction is a potential clinical manifestation of relapsing BCS, and its association with disease progression deserves attention. Clinically, for BCS patients with blood pressure fluctuations, autonomic function assessment should be performed to clarify the etiology and guide targeted intervention.

### 3.4 Monophasic vs. relapsing phenotype of BCS

BCS was traditionally considered a predominantly monophasic inflammatory demyelinating disease with a single acute attack and no relapse (Martinez et al., 2021; Tzanetakos et al., 2020). However, emerging case series and literature reviews have confirmed the existence of relapsing BCS, with an incidence of approximately 15–20% (Etemadifar et al., 2025; Fonseca et al., 2024), and the relapses are mostly accompanied by new clinical symptoms (e.g., limb tremor, autonomic dysfunction) and lesion evolution on MRI (Etemadifar et al., 2025; Mei et al., 2025). Case reports of BCS across age groups—including young adults—have further expanded our understanding of the disease’s clinical heterogeneity (Singh et al., 2024). The relapsing phenotype of BCS is more likely to occur in patients with incomplete initial recovery and uncontrolled vascular risk factors (e.g., untreated hypertension), which is highly consistent with the clinical characteristics of our patient. The difference between monophasic and relapsing BCS may be related to the persistence of inflammatory activity and the status of oligodendrocyte regeneration, and further multicenter studies are needed to identify the predictive factors for relapsing BCS.

### 3.5 Treatment strategies for relapsing BCS

Corticosteroids remain the first-line therapy for acute attacks and relapses of BCS due to their rapid anti-inflammatory effect (Kchaou et al., 2019; Luan et al., 2025). For steroid-refractory relapsing BCS, plasmapheresis and IVIG can be considered as second-line therapies based on individual patient conditions (Elias et al., 2025; Suzuki et al., 2023). At present, the application of disease-modifying therapies (DMTs) in relapsing BCS is still in the exploratory stage: case reports have shown that alemtuzumab and ofatumumab may have a therapeutic effect on relapsing BCS (Elias et al., 2025; Suzuki et al., 2023), but there is no randomized controlled trial to confirm their efficacy and safety. The timing of treatment escalation for relapsing BCS is recommended to be considered in patients with: (1) more than 2 relapses within 2 years; (2) relapse accompanied by progressive motor disability, limb tremor or autonomic dysfunction; (3) continuous lesion evolution on follow-up MRI. For such patients, individualized DMT selection can be attempted on the basis of excluding contraindications.

### 3.6 Prognostic impact of vascular comorbidities and autonomic dysfunction in BCS

Vascular risk factors (e.g., hypertension, diabetes) and autonomic dysfunction are important factors affecting the prognosis of BCS patients. Long-term uncontrolled hypertension can: (1) reduce the blood supply of oligodendrocytes, inhibit oligodendrocyte regeneration and myelin repair, leading to incomplete initial recovery and increased relapse risk; (2) induce cerebral small vessel disease, which synergizes with demyelinating lesions to aggravate motor dysfunction and limb tremor; (3) increase the risk of residual motor disability by affecting the blood–brain barrier function and aggravating central nervous system inflammation. Autonomic dysfunction, as an important manifestation of disease progression, may indicate more extensive involvement of the nervous system, and patients with autonomic dysfunction may have a higher risk of poor prognosis. For BCS patients with vascular risk factors and autonomic dysfunction, strict blood pressure control (target <140/90 mmHg), regular autonomic function assessment and vascular risk assessment are recommended to improve the long-term prognosis.

### 3.7 Clinical implications and follow-up strategy for relapsing BCS

This case underscores the necessity of long-term, standardized follow-up for patients with relapsing BCS. Based on clinical practice and existing evidence, we propose a structured follow-up strategy focused on vascular risk factor management, autonomic function monitoring, and motor function assessment. Vascular risk management includes monthly blood pressure monitoring for the first 6 months post-relapse (transitioning to quarterly thereafter), strict blood pressure control (target <140/90 mmHg) with individualized antihypertensive therapy, and annual evaluation of blood lipids, blood glucose, and cerebral vascular status. Autonomic function should be assessed annually (including blood pressure variability detection and gastrointestinal function evaluation) to enable early identification and timely intervention of autonomic dysfunction. Imaging surveillance involves brain MRI (T1w, T2w, FLAIR, and gadolinium-enhanced T1w) at 6, 12, and 24 months post-relapse, with immediate reimaging if new neurological symptoms (e.g., limb weakness, numbness, tremor, or autonomic dysfunction manifestations) emerge. Motor function assessment entails neurological examinations every 3 months, incorporating MRC muscle strength grading, tremor severity evaluation, ataxia testing, and gait analysis to monitor disease activity and residual motor disability. Additionally, regular follow-up on steroid tapering regimens and personalized motor rehabilitation training (e.g., strength, balance, and gait training) should be provided, with early intervention for new-onset motor symptoms and autonomic dysfunction to minimize the risk of permanent disability. From a therapeutic perspective, the efficacy of repeated corticosteroids for acute relapse is reaffirmed (Suzuki et al., 2023; Kamari et al., 2023a; Kamari et al., 2023b; Jolliffe et al., 2021). However, the residual motor disability despite treatment underscores the limitations of our current approach, and the question of whether early immunomodulation combined with vascular risk factor control and autonomic function intervention could alter long-term outcomes remains open and merits further investigation.

## 4 Limitations and future directions

This study has several limitations that need to be acknowledged. First, the single-case nature limits the generalizability of the conclusions, and the clinical characteristics and treatment experience of this patient cannot be directly extended to all relapsing BCS patients. Second, histopathological confirmation was not performed for the patient, although the diagnosis of BCS was confirmed by the highly characteristic concentric ring-like enhancement on MRI and the typical clinical course. Third, the follow-up period (1 year) is relatively short, which precludes definitive conclusions about the long-term prognosis of relapsing BCS and the natural course of motor symptom recovery in patients with uncontrolled hypertension and autonomic dysfunction. Fourth, detailed dynamic monitoring data of blood pressure and inflammatory factors during the disease course were not collected, which limits the in-depth analysis of the quantitative relationship between hypertension, autonomic dysfunction and BCS inflammatory activity. Fifth, formal neuropsychological assessments (e.g., MoCA, MMSE) were not performed, which limits the comprehensive evaluation of the patient’s cognitive function. However, based on the patient’s clinical manifestations and follow-up observations, there is no evidence of cognitive impairment in this case. Future research directions for BCS are as follows: First, it is urgent to establish international multicenter prospective registries to collect clinical data of relapsing BCS patients, define the incidence of relapsing courses and identify predictive factors for poor outcomes (e.g., uncontrolled hypertension, autonomic dysfunction, incomplete initial recovery, persistent MRI lesion enhancement). Second, randomized controlled trials are needed to evaluate the efficacy and safety of maintenance immunosuppressive therapy and DMTs in preventing BCS relapse, so as to form unified clinical treatment guidelines. Third, longitudinal clinical studies should be carried out to clarify the impact of strict vascular risk factor control (e.g., antihypertensive therapy) and autonomic function intervention on the relapse rate and motor function recovery of BCS patients, and to formulate personalized vascular management and autonomic function intervention strategies for BCS patients with hypertension and autonomic dysfunction. Fourth, further basic and clinical studies are needed to explore the molecular mechanism of hypertension and autonomic dysfunction exacerbating BCS inflammatory demyelination, and to identify potential therapeutic targets for relapsing BCS combined with vascular comorbidities and autonomic dysfunction.

## 5 Conclusion

This case recasts BCS as a potentially relapsing disorder with meaningful long-term motor consequences, and highlights the unrecognized synergistic effect of uncontrolled hypertension and autonomic dysfunction on disease progression and relapse. While corticosteroids remain effective for acute management of relapsing BCS, the specter of relapse and accumulating motor disability demands a paradigm shift toward prolonged monitoring and personalized rehabilitation strategies centered on vascular risk factor control and autonomic function management. Recognizing the full clinical spectrum of BCS—the impact of vascular comorbidities and autonomic dysfunction on the full clinical spectrum of BCS—is essential for optimizing patient care and outcomes in patients with 1-year total follow-up (6 months of stable condition after relapse). For relapsing BCS patients, especially those with uncontrolled hypertension and autonomic dysfunction, prolonged monitoring with a structured follow-up strategy (including strict blood pressure control, regular autonomic function assessment, regular MRI recheck and personalized motor rehabilitation training) is crucial to reduce the risk of further relapse and improve long-term motor functional outcomes.

## Data Availability

The original contributions presented in the study are included in the article/supplementary material, further inquiries can be directed to the corresponding authors.

## References

[ref1] Ananya B. Veeraraghavan V. Kavitha S. Selvaraj J. Gayathri R. (2022). Knowledge and awareness on Balo’s disease among dental students: a survey. J. Adv. Pharm. Technol. Res. 13, S335–S341. doi: 10.4103/japtr.japtr_336_22, 36643112 PMC9836128

[ref2] Anschel D. J. (2006). Reply to the paper by Wiendl et al.: diffusion abnormality in Balo’s concentric sclerosis: clues for the pathogenesis. Eur. Neurol. 55, 111–112. doi: 10.1159/000092788, 16636562

[ref3] Elias S. Hardy T. A. Khan A. Redgrave J. Hoggard N. Coley S. . (2025). Balo’s concentric sclerosis successfully treated with alemtuzumab: long-term follow-up. Mult. Scler. 31, 1600–1602. doi: 10.1177/13524585251331536, 40219936

[ref4] Etemadifar M. Aghili A. Shojaei S. Alaei S. A. Salari M. Norouzi M. (2025). Balo concentric sclerosis, an emerging variant of multiple sclerosis: a case-series and literature review. J. Neuroimmunol. 400:578527. doi: 10.1016/j.jneuroim.2025.578527, 39842344

[ref5] Fonseca A. Santos E. Taipa R. (2024). Balo concentric sclerosis: literature review and report of two cases. J. Neuroimmunol. 392:578370. doi: 10.1016/j.jneuroim.2024.578370, 38797061

[ref6] Jolliffe E. A. Guo Y. Hardy T. A. Morris P. P. Flanagan E. P. Lucchinetti C. F. . (2021). Clinical and radiologic features, pathology, and treatment of Balo concentric sclerosis. Neurology 97, e414–e422. doi: 10.1212/WNL.0000000000012230, 34011576 PMC8362356

[ref7] Kamari C. Galanakis E. Raissaki M. Briassoulis G. Vlachaki G. Vorgia P. (2023a). Correction to: pediatric tumefactive multiple sclerosis case (with Balo-like lesions), diagnostic and treatment challenges. Neurol. Sci. 44:1137. doi: 10.1007/s10072-022-06502-0, 36171523 PMC9816224

[ref8] Kamari C. Galanakis E. Raissaki M. Briassoulis G. Vlachaki G. Vorgia P. (2023b). Pediatric tumefactive multiple sclerosis case (with Balo-like lesions), diagnostic and treatment challenges. Neurol. Sci. 44, 343–345. doi: 10.1007/s10072-022-06396-y, 36171523 PMC9816224

[ref9] Kchaou M. Nagi S. Echebbi S. Ben Ali N. Fray S. Jamoussi H. . (2019). MRI study of Balo’s concentric sclerosis before and after corticosteroid therapy. Rev. Neurol. 175, 327–329. doi: 10.1016/j.neurol.2018.06.010, 30948263

[ref10] Kira J. I. (2025). Treating Balo’s concentric sclerosis in the monoclonal antibody era. Mult. Scler. 31, 1603–1604. doi: 10.1177/13524585251331551, 40219942

[ref11] Luan Z. Habiba K. Z. Sim A. H. Narvaez-Correa I. V. (2025). Balo concentric sclerosis: a rare variant of multiple sclerosis with excellent response to early steroid treatment. Cureus 17:e86059. doi: 10.7759/cureus.86059 40666549 PMC12262001

[ref12] Martinez H. R. Rodriguez-Gonzalez I. C. Escamilla-Garza J. M. Figueroa-Sanchez J. A. Garcia-Aleman A. C. Hinojosa-Gonzalez D. E. (2021). Balo’s concentric sclerosis with monophasic course: a report of 2 cases. Ann. Med. Surg. 68:102602. doi: 10.1016/j.amsu.2021.102602, 34401123 PMC8347801

[ref13] Mei Q. Jiang W. Chen Q. Huang D. Wu L. (2025). An intriguing overlapping: AQP4-IgG-positive neuromyelitis optica spectrum disorder coexisting with Balo concentric sclerosis. J. Neurol. 272:431. doi: 10.1007/s00415-025-13182-1, 40442507

[ref14] Nouri H. Mirmosayyeb O. Badihian S. Shaygannejad V. (2022). Radiologically isolated syndrome: an atypical presentation of Balo’s concentric sclerosis in a patient with the Meniere's disease. Neurol. India 70, 439–440. doi: 10.4103/0028-3886.338680, 35263943

[ref15] Peraza H. Rees J. Kresak J. Montalvo M. Rempe T. (2025). Balo’s concentric sclerosis: a retrospective case series. Mult. Scler. Relat. Disord. 103:106712. doi: 10.1016/j.msard.2025.106712, 40946697

[ref16] Sawczynska K. Kaczowka K. Marona M. Gliniak M. Motyl M. Jagiella J. . (2025). Balo’s concentric sclerosis as a rare stroke mimic. Neurol. Neurochir. Pol. 59, 434–437. doi: 10.5603/pjnns.105737, 40600282

[ref17] Sharifi P. Moradi A. Moghadasi A. N. (2024). Fingolimod-associated Balo’s concentric sclerosis in multiple sclerosis: a case report. Clin. Case Rep. 12:e9266. doi: 10.1002/ccr3.9266, 39109309 PMC11300947

[ref18] Singh M. Almusleh A. Mandru G. Sahu S. Akuma O. Akuma C. M. . (2024). A rare case of Balo’s disease in a young adult: a clinical presentation and management. Clin. Case Reports 12:e8520. doi: 10.1002/ccr3.8520, 38344357 PMC10857917

[ref19] Suzuki D. Suzuki Y. Sato D. Kikuchi K. Akasaka M. Nishida A. . (2023). A case of Balo’s concentric sclerosis showing the attenuation of the Balo lesion after ofatumumab treatment: a case report. J. Neurol. Sci. 450:120694. doi: 10.1016/j.jns.2023.120694, 37270900

[ref20] Thomas M. T. Boyko M. E. Hemsath R. Arukala K. S. Collier V. (2024). A case of Balo concentric sclerosis: a multiple sclerosis mimic. Cureus 16:e67076. doi: 10.7759/cureus.67076 39156999 PMC11330278

[ref21] Tzanetakos D. Vakrakou A. G. Tzartos J. S. Velonakis G. Evangelopoulos M. E. Anagnostouli M. . (2020). Heterogeneity of Balo’s concentric sclerosis: a study of eight cases with different therapeutic concepts. BMC Neurol. 20:400. doi: 10.1186/s12883-020-01971-2, 33138795 PMC7604966

[ref22] Wiendl H. Weissert R. Herrlinger U. Krapf H. Kuker W. (2005). Diffusion abnormality in Balo’s concentric sclerosis: clues for the pathogenesis. Eur. Neurol. 53, 42–44. doi: 10.1159/000084264, 15746544

[ref23] Zhang Y. X. Fang G. L. Tang J. L. Lai Q. L. (2024). Balo’s concentric sclerosis with spontaneous remission and favorable prognosis. Heliyon 10:e33386. doi: 10.1016/j.heliyon.2024.e33386, 39021993 PMC11253651

